# Psychometric properties of the Resilience Scale for Adults (RSA) and its relationship with life-stress, anxiety and depression in a Hispanic Latin-American community sample

**DOI:** 10.1371/journal.pone.0187954

**Published:** 2017-11-10

**Authors:** Roxanna Morote, Odin Hjemdal, Patricia Martinez Uribe, Jozef Corveleyn

**Affiliations:** 1 Department of Psychology, Norwegian University of Science and Technology, Trondheim, Norway; 2 Department of Psychology, Catholic University of Peru, Lima, Peru; 3 Department of Psychology, University of Leuven, Leuven, Belgium; Hunter Holmes McGuire VA Medical Center, UNITED STATES

## Abstract

Resilience is a multi-dimensional construct associated with health and well-being. At present, we do not yet have a valid, scientific instrument that is designed to evaluate adult resilience in Spanish-speaking countries and that accounts for family, social and individual components. This study aimed at investigating the construct and cross-cultural validity of the Resilience Scale for Adults (RSA) by combining Confirmatory Factor Analysis (CFA), Multidimensional Scaling (MDS) and Hierarchical Regression models in a Hispanic Latin-American group. A community sample of 805 adults answered the RSA, Spanish Language Stressful Life-Events checklist (SL-SLE), and the Hopkins Symptom Checklist-25 (HSCL-25). First-order CFA verified the six factors structure for the RSA (RMSEA = .037, SRMR = .047, CFI = .91, TLI = .90). Five RSA scales and total score have good internal consistency (scales *α* > .70; total score *α* = .90). Two second-order CFA verified the intrapersonal and interpersonal dimensions of the protector factors of resilience, as well as their commonality and uniqueness with affective symptoms (anxiety and depression). An exploratory MDS reproduced the relations of RSA items and factors at first and second-order levels against random simulated data, thereby providing initial evidence of its cross-cultural validity in a Spanish-speaking group. The Four-steps hierarchical model showed that the RSA scales are the strongest predictors of anxiety and depression–greater than gender, age, education and stressful life-events. Three RSA scales are significant unique predictors of affective symptoms. In addition, similar to findings in diverse cultural settings, resilience is positively associated with age but not with education. Women report higher scores of Social Resources and Social Competence and lower scores of Perception of the Self. In conclusion, this study demonstrates the construct and criterion-related validity of the RSA in broad, diverse and Spanish speaking sample.

## Introduction

Resilience can be defined as positive resources that may be activated in the context of stress to prevent the development of negative mental health outcomes. As mechanisms of protection, resilience increases the likelihood of adaptive responses. In Latin America, research on resilience is scarce and focused on youth or vulnerable groups (e.g., survivors of war, refugees, and victims of sexual violence). In the main, this research is qualitative and provides in-depth analysis of intrapersonal and cultural aspects of resilience [1–3]. The lack of valid psychometric instruments to evaluate adult resilience, and the absence of community-based information on protective mechanisms, precludes the identification of at risk groups, as well as the development of health enhancing interventions in the Latin-American context. Therefore, this study aims to validate the Resilience Scale for Adults (RSA) [4] in a broad Spanish-speaking community sample of Peruvian adults.

### Resilience, concept and models

Clinical researchers have demonstrated the causal connection between risks, stressors and psychopathology [5,6]. In particular, life stress has been consistently associated with psychosocial and physical challenges for young people and adults [7,8]. However, during the past three decades, the mounting evidence of positive adjustment after facing adversities has shifted the research focus to the protective mechanisms that modify the causal relationship between stress and psychopathology [9,10]. Today, the negative effect of life stress on mental health is considered to be potential in the sense that it may occasion negative effects; however, in some instances, resilience may exercise a protective function. Moreover, diverse developmental trajectories (including positive growth) demonstrate the importance of the health enhancing mechanisms of resilience [11,12].

Resilience is not a general or stable characteristic, nor is it solely an outcome of adaptation in individuals or systems [13]. Resilience is a complex process that concretely manifests itself at specific moments in order to face certain circumstances [14]. Resilience is inferred from the dynamic interactions of their components of risk and adaptation [9]; thus, the effective study of resilience examines individual differences in response to specific environmental threats [10,15]. Empirical research has developed two main perspectives in the study of resilience, namely, the protective and the compensatory models. In the former, protective factors may buffer the impact of stress on outcomes of adaptation such as educational, family, professional, social, health and mental health conditions [16–18]. In contrast, in compensatory models, resilience is evaluated as characteristics of the individual, regardless of the stress experienced. Today, researchers agree on the importance of some dimensions of resilience, including positive characteristics of the individual, stable and supportive families, positive social and community networks, and cultural values [19].

### The Resilience Scale for Adults (RSA)

A methodological review of instruments of adult resilience has revealed that the Resilience Scale for Adults is one of the three instruments with adequate psychometric properties, along with the Connor-Davidson Resilience Scale and the Brief Resilience Scale. The RSA is also the most stable scale (test-retest), with high sensitivity to clinical change [20]. Among these instruments, only the RSA evaluates family and social protective factors of resilience [21,22]. The family and social factors are interpersonal resources built upon relationships that are perceived as meaningful supports for facing adversities and stress. The RSA presents a model that goes beyond the individual self-appraisal and inner characteristics to acknowledge the relevance of perceived resources in the environment. This model might be particularly relevant for evaluating protective mechanisms in multicultural contexts such as in Latin America, where social support networks play a crucial role in adaptation and well-being [23].

Based on an extensive review of the relevant literature, the developers of the RSA tested 195 statements related to resilience and presented a reliable instrument with a five scales structure [24]. Subsequent studies improved the reliability of the instrument by reducing the items (from 45 to 37, and eventually to 33). In addition, the Likert scale response format was modified into a semantic differential format that improved the reliability of the measure [25–27].

Subsequent research showed that a six-factor structure of the RSA is a better model fit in non-clinical samples. The original first factor (Personal Competence) split into two intrapersonal oriented factor-scales: Perception of the Self and Planned Future [25]. These factors, together with Social Competence and Structured Style, are the intrapersonal factors of the RSA. The interpersonal factors are Family Cohesion and Social Resources [28]. Afterward, the developers of the instrument investigated whether the protective factors of resilience are more than just the absence of psychopathology. The hypothesis was that the protective factors evaluated by the RSA are not only positive characteristics of mental health on the same underlying dimension of vulnerability and psychopathology. A second order factor analysis demonstrated that intrapersonal RSA factors are part of a continuum along with mental health outcomes (anxiety, depression and negative thinking), although not with the interpersonal ones. Thus, the RSA measures aspects of protection that may be qualitatively different to the absence of psychopathology [22].

Diverse studies have established the convergent validity as well as the clinical utility of the RSA in Norway. The predictive capacity of protective factors of the RSA was confirmed in natural [26] and experimental conditions [29]. Non-clinical and psychiatric participants show distinctive RSA profiles [4]. Resilience is associated with personality, social intelligence [25] as well as with emotional well-being [30]. Its capacity to predict hopelessness [31] and suicidal ideation [32] has been demonstrated. Resilience is associated with employment but not with education [4]. The preference for planning and organization increases with age (Structured Style); women report more Social Resources, while men show higher scores in intrapersonal strength either as Personal Competence or as Perception of the Self [4,31].

### The RSA in different cultural contexts

The Resilience Scale for Adults has been translated in seven different languages and has been tested in both Western and non-Western cultures, thereby providing evidence of the stability of the construct. In this section, we show studies of the metric invariance, construct validity, and the predictive and convergent validity of the RSA in different cultural contexts. At the end of this section we discuss the relevance of translating and validating the RSA in Hispanic Latin America and in Peru.

Cross-cultural studies have confirmed the six-factor structure, metric invariance, and criteria-related validity of the RSA in Belgium and Brazil [33,34]. In a French-speaking Belgian sample, the RSA factors reported strong positive associations with Sense of Coherence (SOC-13) and five of them showed negative associations with depression and anxiety (HSCL-25). The metric invariance of Structured Style was not demonstrated and it was not associated with affective symptoms, thus researchers recommended a cautious interpretation of this particular RSA factor.

In Brazil, the comparable factor loadings between Norwegian and Brazilian samples shows that participants interpret the item’s contents and use the response scale in similar ways. Moreover, in both countries, women scored higher than men in Social Resources, thus showing that some gender differences may be consistent across these cultures. As expected, the RSA factors reported strong positive associations with Sense of Coherence (SOC) and negative associations with depression and anxiety (HSCL-25) (with the exception of Family Cohesion) [34]. Previously, a multidimensional scaling analysis (MDS) explored the conceptual relationships between factors as well as gender differences in the Brazilian sample. In the configuration MDS plot, women are closer to the social factors while men appeared mainly between Perception of the Self and Structured Style [35].

In Italy, Lithuania and South Africa, Confirmatory Factor Analyses supported the six factors structure of the RSA. In the first case, and as expected, the RSA scales showed positive associations with life satisfaction and social connectedness, and negative associations with hopelessness and psychological distress [36]. In Lithuania, the RSA differentiated between clinical and non-clinical samples (higher association with symptoms of anxiety, depression and anger in the clinical group). In addition, its test-retest reliability was confirmed in the nonclinical group. Lithuanian women scored significantly higher than men in Social Resources and Social Competences, as well as in Family Cohesion [37]. In South Africa, the RSA structure was confirmed and the scores were associated with stress and mental toughness in a sample of competitive athletes, thus providing initial support for the use of the scale in sporting contexts [38].

Studies with community samples and associated constructs were conducted in Iran, India, China and Portugal [39–43]. These studies have explored the predictive and convergent validity of the RSA in relation to contextually relevant psychosocial indicators such as the ones used in the present study (i.e. gender, age, education, stressful life events), or in relation to validated instruments such as the Connor-Davidson Resilience Scale.

In conclusion, the study of the RSA factors across diverse cultural settings is promising. Studies indicate consistent evidence of its factor structure, as well as its clinical and contextual validity in both Western and non-Western contexts. Additionally, most of the studies report that women have higher scores in family and social resources and competences, suggesting that some gender differences may be consistent across cultures.

Despite the use of the Resilience Scale of Adults around the world, the psychometric properties and contextual relevance of the RSA with Spanish-speaking groups in Latin America has not been demonstrated. There are two main motivating factors of this study. First, the Resilience Scale for Adults meets important recommendations for conducting research in Latin America. Unlike other models of adult resilience, the empirical and inductive model developed in Norway equates the relevance of intrapersonal aspects of resilience to resources and competences related to social and family spheres. Latin America is a subcontinent characterized by its collective values and networks of support that act as important assets to overcome individual and social distress [44,45]. Therefore, the RSA might evaluate aspects of protection that express particularized elements of Latin American culture.

Second, in the last decade, Peru and other Latin American countries have experienced rapid and vigorous economic growth. However, Latin America has the largest unbalanced distribution of resources in the world [46,47]. Poverty and inequality have strong impacts in education, health and mental health of millions of people in the continent [48]. In this context, human capital is a strong resource for Latin-American communities; therefore, promoting well-being and mental health is a strategy to foster not just economic development in the region but also, human development.

The use of resilience in academic, developmental or governmental institutions requires validated instruments of evaluation. In this study, we will combine three methods of analysis (i.e. confirmatory factor analyses, multidimensional scaling and hierarchical regression models) to test a broad set of hypotheses regarding the internal structure and convergence of the RSA with associated measures of stress and mental health, as well as with relevant socio-demographic variables for the Peruvian context.

## Materials and methods

Participants were recruited between December 2012 and March 2013 through their universities, work and community organizations. They were invited as volunteers and were informed that the study focuses on resilience and mental health. The inclusion criteria were to be Peruvian, to be older than 18 years of age and to have completed elementary education. Eight hundred and forty-four Peruvian adults answered the Resilience Scale for Adults (RSA), Spanish Language Stressful Life-Events Checklist (SL-SLE), as well as the Hopkins Symptom Checklist-25 (HSCL-25). Eight hundred and five participants correctly completed the surveys (response rate 95.37%)

### Materials

#### Resilience Scale for Adults (RSA)

It is a self-report instrument for evaluating six protective dimensions of resilience in adults: (1) Perception of the Self, (2) Planned Future, (3) Social Competence, (4) Family Cohesion, (5) Social Resources, (6) Structured Style [4,24,25]. It is a reliable (Cronbach’s *α* from .67 to .81 and total score .88) and stable (test-retest, Pearson *r* from .73 to .80, and total score .84) instrument, with a semantic differential format [26]. Cross-cultural studies in Brazil and Belgium found that Resilience scales are reliable: Cronbach’s *α* from .56 (Structured Style) to .79 (Family Cohesion) in Brazil, and from .63 (Structured Style and Social Competence) to .74 (Family Cohesion) in Belgium. In these countries, the RSA total score has a reliability of .88 and .84 respectively [33,34]. The RSA has 33 items; item-response ranges from one to seven; higher scores reflect higher levels of protective factors of resilience.

#### The Hopkins Symptom Checklist (HSCL-25)

The HSCL-25 is a self-report instrument that evaluates psychological adjustment (i.e. anxiety, depression and total distress). Item responses range from ‘*not at all’* (1) to *‘extremely’* (4); the intensification of symptoms is represented by higher scale scores [49]. The HSCL-25 is one of the most widely used screening instruments for psychopathology symptoms [50]. The factor validity (CFA, two factors model: RMSEA = 0.059 and SRMR = 0.055) and reliability (total score, *α* = .90; Anxiety, *α* = .81; and Depression, *α* = .86) of the anxiety and depression subscales and total score have been shown in a Peruvian sample [51].

#### Spanish-Language Stressful Life Events Checklist (SL-SLE)

The instrument identifies the number of stressors (i.e. life-events) a person has experienced throughout their adult life. Participants responses are scored 1 (had been experienced) or 0 (had not been experienced) to each item; the total score ranges from 0 to 20. A higher number of events reflects an increase in life stress. The SL-SLE contains relevant events of diverse domains of adult life. In Peru, 93% of the adults report 0 to 8 events; the increase of life-events is positively associated to the increase of anxiety and depression [51].

#### Statistical analyses

Two linguists and certified translators (English-Spanish) revised the translated RSA protocol in Peru. Descriptive and inferential analyses were completed using SPSS 22. Seventeen RSA item scores were reversed. Eight hundred and forty-four adults answered the RSA and HSCL-25 protocols. First, we eliminated 33 participants with three or more missing responses in the HSCL-25 protocols (10% of the total number of items). The mean score of the item replaced the missing response (one or two missing items) in fourteen HSCL-25 protocols. Then, following the recommendations of the RSA authors [22], participants with more than 10% missing responses in the RSA were removed (four participants). We used the Little's Missing Completely at Random (MCAR) Test to verify that the missing responses in seventy-three RSA protocols were completely at random (Chi-Square statistic = 1414.016, DF = 1133, Sig. = .629). The missing responses (one to three items) were replaced with the mean score for the subscale that the item belonged to. The total number of participants with complete protocols was eight hundred and five. Then, the conditions allowing the inferential analyses were verified (inter-scale correlations, multicollinearity and homoscedasticity).

Confirmatory factor analyses [52] tested the hypothesized latent model using MPLUS7.4 [53]. The re-scaled Satorra-Bentler chi-square statistic (*S-B χ2*) with Robust Maximum Likelihood (MLM) estimation was used to assess the model fit. The *S-B χ2* is a goodness-of-fit indicator that shows the ability of the hypothesized model to reproduce the sample correlation matrix. This is the recommended alternative estimation method for continuous non-normal variables. The criteria of Hu and Bentler [54] were also considered in the analysis: Root Mean Square Error of Approximation (RMSEA) < .06, Standardized Root Mean Square Residual (SRMR) < .08, Comparative Fit Index (CFI) and Tucker-Lewis Index (TLI) ≥ .90., and the lowest Akaike Information Criteria (AIC) for compared models.

International publications have used Exploratory Factor Analysis (EFA) in the developmental phase of the measurement model for the RSA [24,4]. If a measurement model is established, CFA is a stringent test to confirm or disconfirm such a model. In the present study, the CFA is used to confirm and compare different models due to the first use of the RSA in a Spanish-speaking context. At first-order level, the RSA established six factors model [26] was compared to a preceding five factors model [25]. We also compared the six factors model with a two factors model of intrapersonal and interpersonal elements, as well as to a one factor model in which all of the indicators are thought to measure one underlying common factor.

Second-order CFA explored two hypotheses. First, we tested whether the six RSA factors correspond to two latent constructs of intrapersonal and interpersonal elements [28]. Second, we tested if the six RSA resilience factors are, or are not, the exact counterpart of psychopathology symptoms (anxiety and depression). In this case, the hypothesis is that the intrapersonal factors of resilience share common elements with anxiety and depression while interpersonal factors of resilience outline an independent second-order factor. We compared the model of two second-order factors with a one-dimensional model of resilience, anxiety and depression. The second-order models of resilience, anxiety and depression were built upon 58 indicators (33 RSA items and 25 HSCL items), eight first order factors (six of resilience and two of affective symptoms), and one or two second-order factors [22].

Then, the first and second-order structures of the RSA were explored in a non-metric multidimensional scaling analysis (MDS). MDS tests the model fit assuming the monotonic association of items and their non-normal distribution [55]. The input data were Spearman correlations between items and across participants (the matrix of correlations was transformed into dissimilarities). The non-metric stress for the MDS solution was compared with the stress for a random simulation (10,000 replications). The software used was R Studio (package SMACOF).

Finally, criterion-related validity of the RSA was tested, first with correlation analyses of the variables and covariates selected, and then with hierarchical regression models, with depression and anxiety as dependent variables. Based on the literature review, age, gender, education and life-stress were selected as relevant variables that might influence or surpass the capacity of resilience to predict anxiety or depression. Birthplace (or migration) was included because of its relevance in the Peruvian context. The hierarchical linear regression analyses tested the predictive capacity of RSA factors and total score after controlling for gender and age (step 1), education (step 2) and stressful life-events (SL-SLE, step 3). Resilience Total Score and subscales were added in step 4, first as separated scores (7 independent models), and then the six factor scores were entered together in order to compare the unique predictive capacity of each RSA factor (standardized *β* weights) (1 comparison model). Therefore, 8 models were tested for each depend variable. Additionally, the interaction of resilience and stressful life-events (SL-SLE) was tested.

#### Ethics statement

The Doctoral Supervisory Committee of the Faculty of Psychology and Educational Sciences of the University of Leuven (Belgium) approved the research design, selection of instruments and sampling process. The committee included the developer of the RSA as a scientific consultant. Participants did not experience any harm, their anonymity and confidentiality was protected and their written informed consent was obtained. Participants received the contact information of the first author and they were allowed to discontinue their participation during the data collection process.

## Results

Table 1 presents the demographic characteristics of the population studied. The participants form a convenience sample that aims to reflect the diversity of the adult population living in the capital of Peru. Participants are mostly non-migrants in the city of Lima, although one third of the group was born in an inland region of Peru. The group includes men and women with diverse levels of education.

**Table 1:** Demographic characteristics of the study population (N = 805). Partial n may vary due to missing responses.

Characteristics	
Age, years	
Range	18–74
Mean (SD)	28.99 (10.88)
Gender, n (%)	
Female	477 (59.82)
Male	320 (40.20)
Place of birth (n, %)	
Lima	543 (67.45)
Regions	262 (32.54)
Education (n, %)	
Secondary or technical	82 (10.40)
Undergraduate	490 (61.90)
Postgraduate	219 (27.70)

### Confirmatory factor analyses: First and second-order hypotheses

Table 2 summaries the first and second-order confirmatory factor analyses (absolute and comparative fit-indexes). The scaling correction factor (> 1) confirmed the multivariate non-normal distribution and leptokurtosis of the factor indicators, and the increase of the corrected *S-B χ*^*2*^.

**Table 2:** Goodness-of-fit indicators of first and second-order confirmatory factor analyses of the resilience scale for adults and the hopkins symptom checklist -25 (N = 805).

Models^a^			*S-B χ*^*2*^	*Df*	Scaling correction factor	RMSEA	RMSEA CI 95%^a^	SRMR	AIC	CFI	TLI	Regression weights range^b^
RSA—First order												
	1	One Factor	2499.699	495	1.314	.071	.068 - .074	.072	90721	.661	.639	.145 - .604
	2	Two Factors	2049.125	494	1.314	.063	.060 - .065	.067	90130	.737	.719	.168 - .661
	3.	Five Factors	1244.800	485	1.310	.044	.041 - .047	.059	89087	.872	.860	.137 - .850
	4.	Six Factors	1166.470	480	1.309	.042	.039 - .045	.057	88993	.884	.872	.136 - .854
RSA—Second order												
	5.	Intra vs. Interpersonal	1111.458	486	1.310	.040	.037 - .043	.053	88914	.894	.885	.132 - .847
RSA—HSCL Second order												
	6.	Single Factor	3171.604	1582	1.253	.035	.034 - .037	.058	123287	.863	.857	.131 - .848
	7.	Two Factors	2125.521	1581	1.252	.035	.033 - .037	.056	123231	.867	.861	.130 -.852

Resilience Scale for Adults (RSA), Hopkins Symptom Checklist -25 (HSCL). Re-scaled Satorra-Bentler chi-square (S-B χ^2^) for Maximum Likelihood and degrees of Freedom (*df*), Root Mean Square Error of Approximation (RMSEA), Standardized Root Mean Square Residual (SRMR), Akaike Information Criterion (AIC), Comparative Fit Index (CFI), Tucker Lewis Index (TLI)

^a^ Models without the specification of error co-variances

^b^ Standardized

At first-order level (33 items indicators, first four models in Table 2), the six factors structure shows better absolute and comparative fit indexes than the one, two or five factors models. Moreover, the one and two factors models do not reach acceptable model fit indexes (relative or absolute), thereby suggesting that the specification of the protective factors is required in the models. The five and six factors models show good RMSEA, SRMR (below .06 and .08 respectively), and good comparative incremental fit indexes (CFI, TLI ≥ 0.90) [54]; however, the six factors model had slightly better values and the lowest AIC, thus suggesting that it is a better model fit. *S-B* Chi-square values and factors covariance were significant for the first and second-order models shown in Table 2 (*p* < .001).

After first-order models’ comparison, we added two specifications to the six factors model: the co-variance among error terms associated with four observed indicators (item Sc21 with item Sc15, and item Ps13 with item Pf14). These modifications are minor changes, and both are theoretically justifiable because the items belong either to the same factor (Social Competence) or to the intrapersonal dimension (Perception of the Self and Planned Future). The final fit indexes of the RSA six factors model in Peru are RMSEA = .037, SRMR = .047, CFI = .91, TLI = .90.

At second-order level, model five confirms the bi-dimensionality of the protective factors of resilience (interpersonal and intrapersonal dimensions). Models six and seven test the hypothesized commonality vs. uniqueness of resilience and psychopathology (eight factors indicators for second-order analysis) [22]. Both models show good global model fit indexes and significant factor loadings (*p* < .001, two-tailed). Model six shows the expected negative loadings of anxiety (*λ* = -.73) and depression (*λ* = -.78) with the single second-order factor. However, the slightly better model fit indexes and the lowest AIC points towards bi-dimensional model seven as a marginally better model. In model seven (two second-order factors), the intrapersonal second-order factor has significant loadings with Perception of the Self (*λ* = .92), Planned Future (*λ* = .82), Social Competence (*λ* = .70), Structured Style (*λ* = .63), Anxiety (*λ* = -.75) and Depression (*λ* = -.80). Factor loadings of Anxiety and Depression are stronger than in model six. As expected, Family Cohesion and Social Resources comprise the interpersonal second-order factor (*λ* = .75 and *λ =* .85, respectively). The covariance between the two second-order factors is significant (*σ*^*2*^_*xy*_ = .79).

In the seven models tested, item factor loadings are significantly different from 0 (*p* < .001, two-tailed). All items are significantly negatively skewed (Shapiro-Wilk *p* < .001) and their kurtosis is non-normal (Z > 1.96, *p* < .05).

In the Peruvian community sample, RSA total scale and five subscales show good internal consistency (Cronbach's alpha) [56,57]: Perception of the Self *α* = .78, Planned Future *α* = .71, Social Competence *α* = .70, Family Cohesion *α* = 80, Social Resources *α* = .76, Structured Style *α* = .48. The complete RSA scale has a Cronbach's alpha of .90 (with no items suggested to be deleted to increase this value).

**Fig 1:**
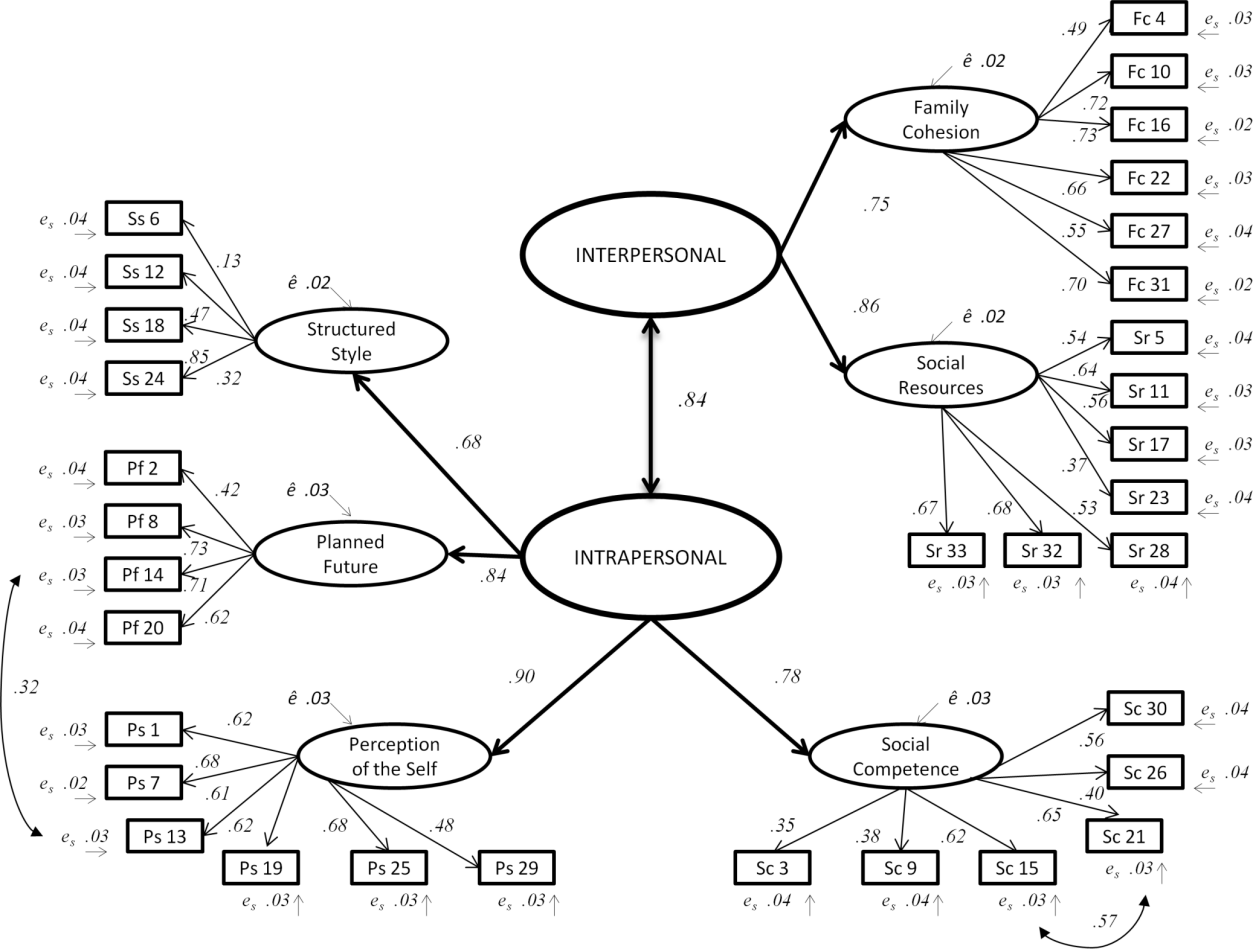
Second-order confirmatory factor analysis of the resilience scale for adults (N = 805).

Fig 1 shows the second-order model of the RSA with its Intrapersonal and Interpersonal dimensions. The estimates reported in Fig 1 are standardized and significant at *p* < .001 (two-tailed). Thirty-two RSA items (up to 33) show a statistically significant squared multiple correlation (*p = 0*, two-tailed). Item Ss 6 (Structured Style: *'I am at my best when I'*) has a significant *R*^*2*^ at *p* < .1. Special attention will be given to item six in the final section (Discussion). RSA factors covariance (*σ*^*2*^_*xy*_) are significant and strong (*p* < .001, two-tailed).

### Multidimensional scaling analysis

**Fig 2:**
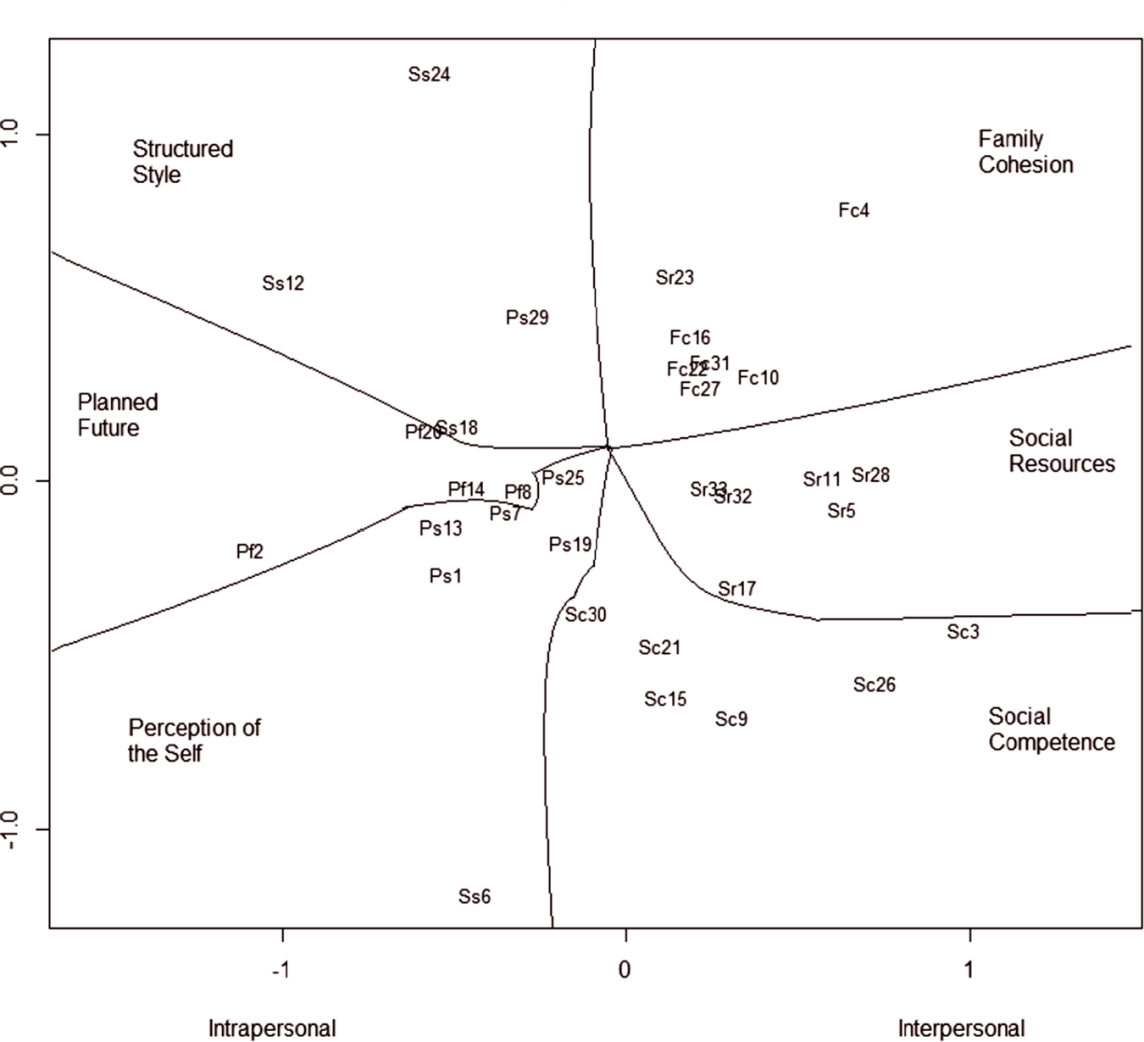
Multidimensional scaling: Six-factors and second order dimensions (N = 805).

A MDS analysis confirms the six-factor structure of the RSA. The general non-metric stress for the MDS solution is .21 in the Peruvian sample. The non-metric stress for simulated random data (10,000 replications) was much higher (.33), indicating that there is a meaningful structure in the data. Thus the MDS configuration plot (Fig 2) is a good representation of the relations between RSA items and factors.

The X axis of the two dimensional configuration plot divides intrapersonal and interpersonal scales. The space is portioned in a polar way in order to separate the six scales. Similar to results reported in Brazil [35], the Euclidean distances between items and their location in a low-dimensional space confirms the bipolarity of interpersonal and intrapersonal dimensions of resilience − with Social Competence as a clear interpersonal factor. Three items show unexpected patterns: items Ss 6 (Structured Style: *'I am at my best when I')*, Ps 29 (Perception of Self: *'Events in my life that I cannot influence')*, and Sr 23 (Social Resources: *'When a family member experiences a crisis/emergency')*. These items showed the highest values in the stress per-point evaluation. However, the three items remain in their expected intra or interpersonal dimension.

### Criterion-related validity of the RSA: Correlation analysis and hierarchical regression models

Table 3 reports means, standard deviations and Pearson’s correlation among all of the variables studied.

**Table 3:** Means, standard deviations and Pearson's correlations between variables studied (N = 805).

	Variables (No. items)	Mean	SD	1	2	3	4	5	6	7	8	9	10	11	12	13	14
1	Gender	.60	.49														
2	Place of birth	1.33	.47	-.02													
3	Age	28.52	10.63	-.02	.21^b^												
4	Education	3.11	.72	.07	-.03	-.11 ^b^											
5	SL–SLE (20)	3.83	2.76	.03	.12 ^b^	.41 ^b^	-.05										
6	Anxiety (10)	1.50	.39	.11 ^b^	-.10 ^b^	-.18 ^b^	-.03	.02									
7	Depression (15)	1.49	.41	.07 ^a^	.00	-.06	-.10 ^b^	.09 ^a^	.71 ^b^								
8	HSCL—25 Total (25)	1.5	.37	.09 ^b^	-.04	-.12 ^b^	-.08 ^a^	.07	.89 ^b^	.96 ^b^							
9	Perception of Self (6)	5.37	1.04	-.09 ^a^	.14 ^b^	.33^b^	-.03	.15 ^b^	-.55 ^b^	-.55 ^b^	-.59 ^b^						
10	Planned Future (4)	5.07	1.10	.03	.03	.20 ^b^	.05	.03	-.39 ^b^	-.47 ^b^	-.47 ^b^	.58 ^b^					
11	Social Competence (6)	5.32	1.00	.08 ^a^	.06	.25 ^b^	-.02	.12 ^b^	-.30 ^b^	-.31 ^b^	-.34 ^b^	.46 ^b^	.38 ^b^				
12	Family Cohesion (6)	5.46	1.10	.02	.12 ^b^	.23 ^b^	-.01	.03	-.31 ^b^	-.36 ^b^	-.37 ^b^	.44 ^b^	.40 ^b^	.36 ^b^			
13	Social Resources (7)	6.00	.81	.17 ^b^	.03	.15 ^b^	.02	.05	-.34 ^b^	-.38 ^b^	-.39 ^b^	.46 ^b^	.41 ^b^	.59 ^b^	.52 ^b^		
14	Structured Style (4)	5.18	1.01	-.01	.11 ^b^	.24 ^b^	-.02	.03	-.23 ^b^	-.26 ^b^	-.27 ^b^	.38 ^b^	.40 ^b^	.27 ^b^	.31 ^b^	.27 ^b^	
15	RSA Total (33)	5.45	.72	.05	.12 ^b^	.33 ^b^	-.01	.10 ^b^	-.50 ^b^	-.55 ^b^	-.57 ^b^ **	.78 ^b^	.71 ^b^	.72 ^b^	.73 ^b^	.77 ^b^	.56 ^b^

Spanish-Language Stressful life events (SL-SLE), Hopkins Symptom Checklist -25 (HSCL). Gender and place of birth are categorical dichotomous variables (male = 0, female = 1; Lima = 0; regions = 1); education is ordinal (0 lowest to 4 highest education); age and SL-SLE are continuous variables. All scales are scored such that higher numbers represent higher levels of the constructs.

^a^ *p* < .05

^b^ *p* < .01 (two-tailed).

Significant positive correlations were found for female participants with the scales of psychopathology symptoms (HSCL Anxiety, Depression and Total score), RSA Social Competences and Social Resources. Perception of the Self was significantly negatively related to female gender. Being born in inland regions of Peru is significantly positively correlated with three RSA factor scales (Perception of the Self, Family Cohesion and Structured Style), RSA Total Score and Stressful life events (SL-SLE). It is also negatively related to Anxiety. All of the protective factors of resilience increase significantly with age. Age is negatively correlated with HSCL Anxiety and HSCL Total Score. In contrast, education is not correlated with the protective factors, although it is negatively significantly related to Depression and HSCL Total Score. As expected, the correlations between the RSA and HSCL-25 scales are negative. Collinearity statistics are all within accepted limits for multiple regression analyses (i.e. Tolerance from .69 to .98, Variance Inflation Factors from 1.25 to 1.98).

Prior to the hierarchical regression analyses, extreme univariate outliers of the two dependent variables, anxiety and depression (HSCL-25) and stress (SL-SLE total score higher than eight), were controlled. Scale scores were log-transformed. The hierarchical multiple regression analyses tested a set of hypotheses regarding the prediction of anxiety and depression by resilience. Table 4 summarizes eight hierarchical regression models for each dependent variable (ie. HSCL Anxiety and Depression). First, the six RSA scales’ scores and Total Score were inserted in the fourth step of the hierarchical model independently (Step 4: RSA independent main effect, seven models); then, the six RSA scales' scores were inserted together to compare the effect of each scale in the fourth step of the model (Step 4: RSA scales compared, one model). The hypotheses were confirmed: RSA Total Score and factors (independently or together) account for the variance of HSCL Anxiety and Depression after controlling for the effect of gender, age (step 1), education (step 2) and Stressful life-events—SL-SLE (step 3).

**Table 4:** Summary of hierarchical regression analyses for resilience scale for adults main effect and factors compared predicting anxiety and depression.

		HSCL Anxiety (*N* = 741)					*HSCL Depression (N = 742)*				
Predictors		*Β (95% CI)*	*β*	*R*^*2*^_*Adj*._	*ΔR*^*2*^	*ΔF*		*Β (95% CI)*	*β*	*R*^*2*^_*Adj*._	*ΔR*^*2*^	*ΔF*
Step 1: Demographics				.05		18.42 ^c^			.01		5.82 ^b^
	Gender	.06 (.02; .09)	.12 ^c^				.05 (.01; .09)	.10 ^b^			
	Age	-.004 (-.006; -.002)	-.18 ^c^				-.01 (-.01; .00)	-.07			
Step2: Education				.05	.01	1.63			.02	.01	3.10 ^a^
	Secondary	.01 (-.07; .10)	.01				.12 (.03; .21)	.10 ^a^			
	Technical	.06 (-.03; .15)	.05				.09 (-.01; .18)	.07			
	Undergraduate	.05 (.01; .09)	.09 ^a^				.04 (-.01; .08)	.07			
Step3: SL- SLE											
	Stressful life-events	.06 (.03; .09)	.14 ^c^	.07	.02	13.63 ^c^	.07 (.04; .11)	.17 ^c^	.05	.03	18.94 ^c^
Step 4: RSA main effect (seven independent models for each dependent variable)											
	RSA Total Score	-.76 (-.86; -.65)	-.46 ^c^	.26	.19	177.43 ^c^	-.97 (-1.08; -.86)	-.55 ^c^	.33	.28	292.41 ^c^
	Perception of the Self	-.52 (-.58; -.45)	-.49 ^c^	.29	.22	220.47 ^c^	-.61 (-.68; -.54)	-.55 ^c^	.33	.28	292.66 ^c^
	Planned Future	-.28 (-.34; -.22)	-.32 ^c^	.16	.10	81.79 ^c^	-.39 (-.46; -.33)	-.43 ^c^	.22	.17	156.62 ^c^
	Social Competence	-.31 (-.39; -.23)	-.27 ^c^	.14	.07	56.17 ^c^	-.38 (-.47; -.30)	-.32 ^c^	.14	.10	80.2 ^c^
	Family Cohesion	-.26 (-.33; -.19)	-.26 ^c^	.13	.07	52.92 ^c^	-.34 (-.41; -.27)	-.33 ^c^	.15	.10	85.63 ^c^
	Social Resources	-.46 (-.56; -.35)	-.30 ^c^	.15	.09	72.80 ^c^	-.58 (-.68; -.47)	-.37 ^c^	.18	.13	110.93 ^c^
	Structured Style	-.21 (-.29; -.13)	-.19 ^c^	.10	.04	27.42 ^c^	-.30 (-.38; -.22)	-.26 ^c^	.11	.06	50.08 ^c^
Step 4: RSA scales compared (one model for each dependent variable)											
				.30	.24	40.10 ^c^			.37	.33	61.10 ^c^
	Perception of the Self	-.42 (-.50; -.33)	-.40 ^c^				-.41 (-.50; -.32)	-.37 ^c^			
	Planned Future	-.04 (-.11; .03)	-.05				-.13 (-.20; -.07)	-.14 ^c^			
	Social Competence	-.07 (-.16; .02)	-.06				-.08 (-.17; -.00)	-.07			
	Family Cohesion	-.05 (-.13; .02)	-.06				-.09 (-.16; -.01)	-.08 ^c^			
	Social Resources	-.08 (-.21; .05)	-.05				-.12 (-.24;. 01)	-.07			
	Structured Style	-.01 (-.08; .07)	-.01				-.04 (-.11;. 04)	-.03			

Resilience Scale for Adults (RSA), Hopkins Symptom Checklist -25 (HSCL). *N* changes due to the control of outliers for HSCL and SL-SLE. Education variables are dummy with 0:‘graduated’ as the reference group. Unstandardized and standardized Beta weights (*β)* of Demographics, Education and SL-SLE belong to the step where they were introduced in the model.

^a^ *p* < .05 (two-tailed).

^b^ *p* < .01 (two-tailed).

^c^ *p* < .001 (two-tailed).

In step one, gender and age were significant predictors of the dependent variables (*R*^*2*^_*Adj*._). Gender was statistically significantly for both Anxiety and Depression while age was significant for Anxiety (final *β* weights). Women and younger participants have more probability of experiencing high levels of symptoms. In step two, the change in variance accounted for by education (*ΔR*^*2*^) was significant for Depression. Secondary education was a statistically significant unique predictor of Depression while Undergraduate education was for Anxiety (final *β* weights). In step three, Stressful life-events (SL-SLE) was a statistically significant predictor of Anxiety and Depression above the demographic characteristics (*ΔR*^*2*^ and final *β* weights).

In step four, the RSA Total Score and 6 RSA subscales were inserted, creating seven independent models for each outcome variable. When comparing the first independent seven models (Step 4: RSA independent main effect), each RSA scale and Total Score significantly explained the variance of Anxiety and Depression above demographic characteristics, education and Stressful life-events (standardized *β* weights, *p* < .001). In these models, it is possible to state with 95% confidence that the magnitude of each unstandardized *β* differs from zero and that they are significant unique predictors of Anxiety and Depression. Perception of the Self is the protective factor that contributes more to the prediction (*ΔR*^*2*^) of the dependent variables. Models with Perception of the Self in the fourth step account for 29% of the total variability of Anxiety and 33% of Depression (*R*^*2*^_*Adj*._).

Finally, the RSA factor scores were added together in the fourth step of an additional model (Step 4: RSA scales compared, eighth models for each dependent variable). The purpose was to compare the independent predictive capacity of each RSA factor (*β* weights). Perception of the Self is a significant predictor for the two dependent variables. Planned Future and Family Cohesion have unique predictive capacity on Depression (standardized *β* weights, *p* < .001). Accordingly, the confidence intervals associated with the unstandardized *β* weights show with 95% of probability that these estimates differ from zero. Together, the six independent RSA scales accounted for the highest percentage of variance of the dependent variables: 30% of Anxiety and 37% of Depression (*R*^*2*^_*Adj*._).

The hypothesized interaction effect of life stress (SL-SLE) and resilience (RSA factors) on the prediction of Anxiety and Depression was not demonstrated. In an additional fifth step, the parameters of change (*R*^*2*^_*Adj*.,_ *ΔR*,^*2*^ *ΔF*) were not significant and *β* weights showed unexpected associations (positive) between the interaction term (resilience x stress) and psychopathology symptoms. The created interaction terms (resilience x stress) had inappropriate levels of Tolerance and VIF showing a high degree of linear dependency among Stressful life events and the RSA scales. Under these conditions, the interaction term cannot be properly used in the model.

## Discussion

The cross-cultural validity of the RSA has been successfully tested with different methods in Brazil (Portuguese speaking) and Belgium (French speaking). The instrument has also been tested and used in Italy, Lithuania, South Africa, Iran, China, and India. For the first time, a study confirms the construct validity of the RSA in a Spanish-speaking group and contributes to the evidence of its cross-cultural validity in Latin America. The confirmatory factor analyses verified the six-factor structure, the intrapersonal and interpersonal dimensions of resilience, and its commonality − and simultaneous − uniqueness from psychopathology (anxiety and depression). The latest results contribute to understanding the concept of resilience as mechanisms of protection that may act beyond their relation to mental health symptoms. As a multidimensional construct, resilience is construed as mechanisms that may foster recovery or positive growth despite the presence of symptoms of anxiety and depression.

In terms of external validity, the six protective factors of resilience explain significant amounts of variance in anxiety and depression above other relevant variables such as life stress, age, gender, and education. The RSA scales are also significantly correlated with demographic characteristics (age, gender, and birthplace) and the increase of stressful life events. Unlike studies in other cultural settings, the RSA scales do not correlate significantly with education. Consequently, the RSA provides contextually relevant information for resilience research in Peru.

To the best of our knowledge, this is the first validation study of the Resilience Scale for Adults in Hispanic Latin America that employs a broad community sample. Recent studies tested other resilience instruments with Spanish-speaking young adults [58], used clinical samples [59–61], or they were not conducted in Latin America [62–64]. The RSA encompasses person-environment interactions because it acknowledges family and social competences and resources of resilience. Our results indicate that the RSA is a suitable instrument for research in non-Western settings where social networks are particularly meaningful in facing adversities.

We demonstrate that Multidimensional Scaling (MDS) approach strongly complements the Confirmatory Factor Analyses of the RSA. MDS is not based on covariance structure analysis and is therefore especially useful to investigate positive constructs in a community sample. MDS is a theory construction method that assumes a metric space between items based on contents. Hence, a certain invariance of the structural hypothesis can be interpreted [55,65]. This is especially relevant because the RSA was developed in a different cultural context than Peru (i.e. Norway), and we therefore present preliminary evidence of the cross-cultural validity of the RSA in a Spanish-speaking Latin American sample.

In the MDS analysis, it is interesting to note that Social Competence items appear between the main intrapersonal and interpersonal factors (Perception of the Self and Social Resources, respectively). The MDS configuration plot depicts Social Competence items closer to Social Resources and Family Cohesion than to the other Intrapersonal factors (i.e. Planned Future and Structured Style). MDS analysis of the RSA in Brazil (Portuguese-speaking) also located the Social Competence items closer to the interpersonal dimension [35]. Although competences are commonly understood as characteristic of the individual, they are closer to the interpersonal factors in the two Latin American samples.

Similar to previous studies, there is some weakness in the sixth RSA factor, Structured Style. In Norwegian samples, Structured Style has the lowest internal consistency (*α* = .67) but one of the highest test re-test reliabilities (*r* = .80) [26]. Good reproducibility (i.e. test-retest reliability above.70) [56] is a key recommended attribute for judging instruments in health service research, clinical care and policy making, especially in considering diverse cultural settings [66]. In Brazil and in Peru, Structured Style had the lowest internal consistency, though the graphic inspection with MDS showed that the factor and its items fit well in the expected model [34,35]. In Peru, Structured Style has stronger inter-scale correlations than those reported in either Norway or Belgium. The CFA and MDS analyses indicate that Structured Style is a meaningful factor of the Resilience Scale for Adults, though it must be used cautiously in making individual assessments in Peruvian groups.

In terms of distance in the MDS graph, Structured Style is an intrapersonal factor closer to Planned Future than to Perception of the Self. The items belonging to this factor have the longest distances in between them. Moreover, the item Ss 6 (Structured Style: *'I am at my best when I')* appears in Perception of Self, opposite from its original factor, and it seems, the least related to the other thirty-two RSA items. Information gathered during the fieldwork might clarify the patterns of response to item Ss6 in Peru. Across different settings (universities, public or volunteer institutions), participants asked for clarifications about item Ss 6. Neither the translated statement nor the polarities of the semantic differential were clearly understood. Apparently, the statement in Spanish should explicitly show the expected efficacy of the behavior, and the semantic differential should make a clear distinction between a structured orientation versus spontaneity to achieve goals. This item should be revised in further studies.

Confirmatory Factor Analysis and Multidimensional Scaling are complementary methods to explore the construct validity of the RSA. Whereas CFA represents the relations between items and latent structures, MDS emphasizes the relationship between items, allowing them to take any location in the two-dimensional space [67]. Consequently, the two analytic approaches enhance our understanding of the RSA structure and allow for further research in similar populations.

The power of the RSA factors-scales to predict anxiety and depression symptoms is substantial (independently, together, or as a total score). In this study, we have analyzed the predictive capacity of each RSA Scale and Total Score, and then, in an additional model, we have compared the predictive capacity of the six RSA factors scales. This way of analyzing the data serves a dual purpose. Firstly, analyzing the individual RSA factors in independent analyses makes it possible to answer the research question if the individual RSA factor predicts levels of anxiety and depressive symptoms. Our results show that each of the RSA factors points to different aspects of resilience and may be used individually. Secondly, rerunning the analysis with all RSA factors in the same step answers the question if any of the RSA factors predict a unique variance that the other factors do not. In the case of Anxiety, the RSA factor Perception of the Self has this capacity above the other RSA factors, while in case of Depression, Perception of the Self, Planned Future, and Family Cohesion has this strength. This analysis illustrates the different aspects of adult resilience related to each RSA factor, and it shows their capacity as mental health predictors in this context.

Moreover, the results are significant and useful considering that this study is based on a community sample of diverse adults. Interestingly, the association of the resilience factors and age confirms that the increase of protective factors of resilience might be an outcome of development [68,69] and it is not an outcome of better educational opportunities in Peru. Gender differences are also consistent with studies conducted in diverse cultural settings. While women show more Social Resources and Competences, men show more resources related to a positive Perception of the Self.

Our results also show that the increase of resilience is associated with larger amounts of life stress and migration. Perception of the Self and Total Score increases in both cases. Social Competence increases in instances of larger amounts of life stress, while Family Cohesion and Structured Style is higher in participants who migrated to Lima. These results may show that protective factors of resilience are more active in adverse life circumstances.

### Limitations

Two important limitations of our study are the cross-sectional design and the way in which we gathered the information of stressful life events (SL- SLE). These two conditions did not allow us to test the hypothesis that the interaction between life stress and resilience is a stronger predictor of anxiety and depression under stress conditions (i.e. protective model of resilience). This hypothesis has been demonstrated under experimental conditions and in prospective studies. We did not specify a controlled period for the occurrence of the life stressors evaluated. The buffering effect of resilience on the impact of life stress has been demonstrated in research designs that evaluate recent stressors (i.e. one year or less). We suggest that in natural contexts, research designs should control the time elapsed from the occurrence of the stressor and the evaluation of protective factors and outcomes.

### Further research

Further research should evaluate the metric and measurement invariance (multiple-group CFA) of the RSA in community samples of Peru and Norway. As demonstrated in Europe and Brazil [33,34], RSA latent constructs are relevant and comparable across diverse cultural contexts. Longitudinal or experimental studies should test the interaction effect of RSA factors and stress. Although the evaluation of stress in natural contexts is challenging, information about changes in stress experienced should demonstrate the buffering effect of the protective factors of resilience in psychopathology outcomes. The structural validity of the RSA in a broad and diverse sample is a promising step in these processes.

### Conclusion

The validation of the RSA will allow for uncovering and understanding the complex paths of resilience, to track changes in life trajectories and to compare contexts and outcomes where resilience may be expressed as positive growth [28]. The RSA evaluates adult resilience with a comprehensive perspective, including family and social resources. In line with updated theory, the RSA is a psychometric tool that evaluates protective factors in a non-person centered perspective, and therefore, it is especially relevant in cultural contexts in which social connections are basic supports and highly influential in determining one’s identity. Moreover, the RSA is recommended for research on public policy and interventions design [70], thus the valid use of the RSA, in connection with measurements of psychopathology or indicators of psychosocial adjustment may enhance research for public policies directed towards vulnerable groups. The RSA might also be used to further uncover unexpected paths of adaptation in harsh contexts, especially when social bonds are disrupted (e.g. natural or human-made disasters, excluded groups, etc.). A valid and culturally relevant instrument of resilience will enhance mental health prevention, promotion, and interventions, and it will allow for an improvement in individual and community well-being in Hispanic Latin America, particularly in Peru.

## Supporting information

S1 Dataset (SAV)
