# Correction: Psychometric properties of the Resilience Scale for Adults (RSA) and its relationship with life-stress, anxiety and depression in a Hispanic Latin-American community sample

**DOI:** 10.1371/journal.pone.0196139

**Published:** 2018-04-16

**Authors:** The PLOS ONE Staff

The Funding section is incorrect. The correct funding information is: RM received funding from the Norwegian University of Science and Technology and from the Catholic University of Peru (fieldwork: ID364). The publisher apologizes for the error.
